# Cardiac tamponade due to eosinophilia treated with intravenous corticosteroid: A case report

**DOI:** 10.1097/MD.0000000000034410

**Published:** 2023-08-04

**Authors:** Hiroaki Taniguchi, Mayuko Kaneko, Nobuaki Kiriu, Tetsuro Kiyozumi

**Affiliations:** a Department of Traumatology and Critical Care Medicine, National Defense Medical College, Saitama, Japan.

**Keywords:** cardiac tamponade, corticosteroid, eosinophilia

## Abstract

Cardiac tamponade, a condition in which the heart is compressed by pericardial fluid retention, is easy to diagnose; however, identifying the cause may be challenging since it can be caused by a variety of conditions, including trauma and pericardial disease.

**Patient concerns:**

A 22-year-old man was admitted to the intensive care unit with respiratory failure. He had previously received allogeneic hematopoietic stem cell transplantation for acute myeloid leukemia and developed chronic graft-versus-host disease (cGvHD) that was treated with a corticosteroid. At this time, he developed bilateral femur head necrosis and underwent surgery after discontinuation of the corticosteroid but developed respiratory failure postoperatively. The initial diagnosis was cardiac failure, which temporarily improved with treatment; however, eosinophilia and pericardial effusions became prominent.

**Diagnoses:**

Pericardial effusion gradually progressed, resulting in cardiac tamponade.

**Interventions:**

Pericardiocentesis was performed. Eosinophilia could be the cause of cardiac tamponade; thus, corticosteroid was administered.

**Outcomes:**

Pericardial effusion improved remarkably after corticosteroid administration. The corticosteroid dose was gradually tapered, and the patient was discharged.

**Lessons:**

This case presented with cardiac tamponade associated with eosinophilia, probably owing to graft-versus-host disease. This is an unusual condition associated with a history of hematologic neoplasms; although evaluation is challenging, appropriate assessment could help save the patient’s life.

## 1. Introduction

Cardiac tamponade is a condition in which the heart is compressed by pericardial fluid retention. It can occur as a result of pericardial disease, trauma, or cardiac rupture and is life-threatening.^[1]^ Electrocardiography and echocardiography are important diagnostic tools.^[2]^ Treatment depends on the cause; however, pericardiocentesis is performed in emergencies, such as cases of patients with hypotension. We report a case of cardiac tamponade associated with eosinophilia that required urgent pericardiocentesis; however, the cause was unclear, and definitive treatment could not be promptly initiated.

## 2. Case presentation

A 22-year-old man was admitted to the intensive care unit for respiratory failure. He had a history of acute myeloid leukemia, which was treated with allogeneic hematopoietic stem cell transplantation. Thereafter, he developed chronic graft-versus-host disease (cGvHD) and was administered a corticosteroid. He also developed anthracycline-induced cardiomyopathy and chronic renal failure requiring peritoneal dialysis. At this time, he developed bilateral femur head necrosis and underwent left hip replacement surgery; however, his respiratory status worsened postoperatively. This led to him being examined by our department.

**Figure 1:**
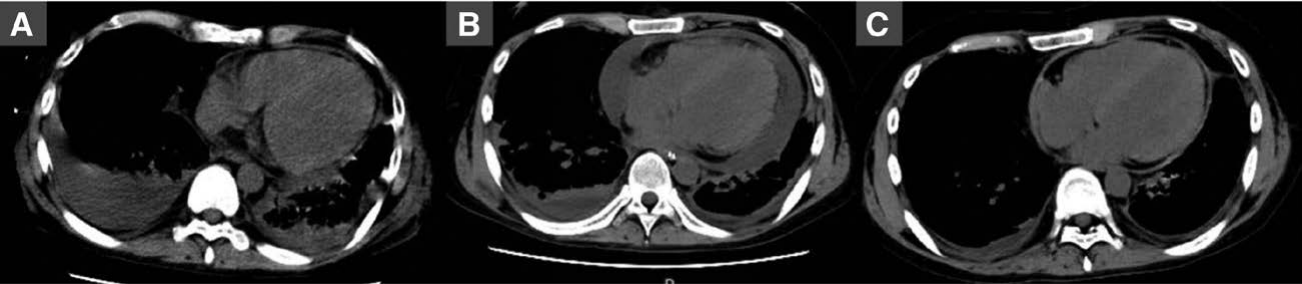
. A chest computed tomography (CT) scan performed after admission to the intensive care unit (A), on the 39th d (B), and the 57th d (C). (A) CT showed pericardial effusion and pleural effusion. (B) Pericardial fluid increased. (C) Pericardial effusion improved.

Initial physical examination revealed a Glasgow Coma Scale score of E1V1M1. The heart rate was 150 beats/min, and the blood pressure was 189/154 mm Hg. The respiratory rate was 30 breaths/min. Arterial blood gas analysis revealed metabolic and respiratory acidosis, and the initial blood test results revealed inflammation, respiratory failure, and heart failure (Table 1). Chest computed tomography (CT) performed after admission to the intensive care unit (Fig. 1A) revealed pericardial and pleural effusion.

**Table 1:** Blood test at admission to the intensive care unit.

Variables	Results
Arterial blood gas analysis*	
pH	7.035
PaCO_2_ (mm Hg)	77.5
PaO_2_ (mm Hg)	80.6
HCO_3_ (mmol/L)	19.7
Base excess (mmol/L)	-11.5
Lactate (mmol/L)	4.5
Complete blood cell count	
White blood cell count (×10^3^/μL)	18.5
Neutrophil percentage (%)	63.2
Lymphocyte percentage (%)	28.0
Eosinophil percentage (%)	3.4
Red blood cell count (×10^6^/μL)	3.28
Hemoglobin (g/dL)	9.5
Platelet count (×10^4^/μL)	47.8
Blood biochemistry	
Total bilirubin (mg/dL)	0.71
AST (U/L)	29
ALT (U/L)	7
LD (U/L)	576
ALP (U/L)	147
BUN (mg/dL)	21
Creatine (mg/dL)	3.37
Sodium (mmol/L)	137
Potassium (mmol/L)	3.8
Chlorine (mmol/L)	105
C-reactive protein (mg/dL)	19.1
Brain natriuretic peptide (pg/mL)	2070
Troponin I (pg/mL)	71.4
Blood coagulation	
Activated partial thromboplastin time (s)	35.5
PT activity (%)	72.9

ALP = alkaline phosphatase, ALT = alanine aminotransferase, AST = aspartate aminotransferase, BUN = blood urea nitrogen, LD = lactate dehydrogenase, PT = prothrombin time.

* Blood gas analysis was performed under oxygen administration at 15 L/min using a mask with a reservoir.

**Figure 2:**
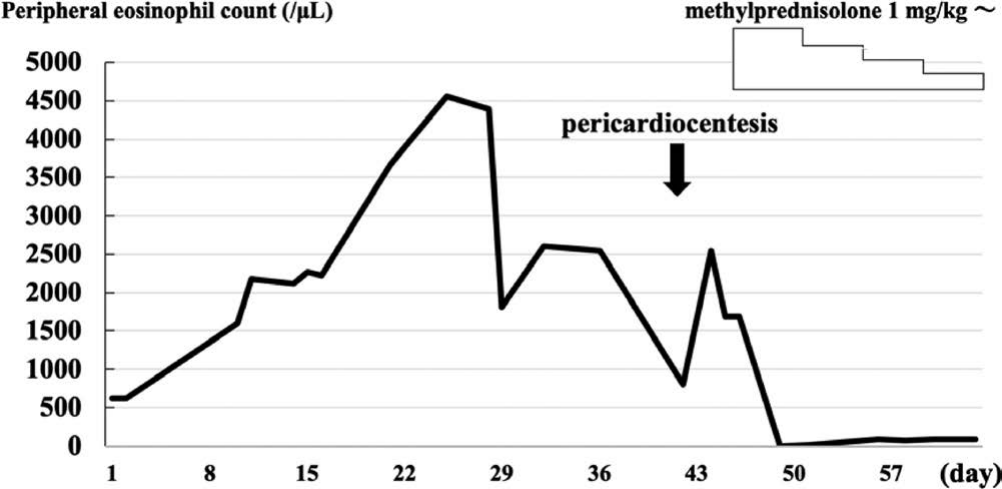
. Peripheral blood eosinophil counts are shown as a solid line. Dramatic improvement was observed with the initiation of methylprednisolone.

The patient was initially diagnosed with acute heart failure and pneumonia and was placed on a mechanical ventilator. Antibiotics and medications for heart failure were administered. Eosinophilia was detected (Fig. 2), and laboratory tests were performed to identify the cause, including autoimmune and neoplastic diseases. Weaning from the ventilator was attempted but was challenging, and a tracheostomy was performed on day 11. Respiratory failure persisted, and computed tomography was performed on day 39, which showed increased pericardial effusion (Fig. 1B). In contrast, the blood test results revealed no increase in the cardiac biomarkers, and no electrocardiographic changes characteristic of pericarditis were observed. On day 42, the pulse pressure decreased, dyspnea worsened, and echocardiography revealed increased pericardial effusion, resulting in a diagnosis of cardiac tamponade; pericardiocentesis was subsequently performed. The puncture fluid was dark red (700 mL), and examination revealed a high cell count of 1507 cells/μL (75.5% neutrophils, 16.5% lymphocytes, and 6% eosinophils). The bacterial culture was negative, and no malignant findings were observed. After pericardiocentesis, the symptoms temporarily improved; however, the pericardial fluid refilled. Since eosinophilia was suspected to be the cause of pericardial effusion, intravenous corticosteroid (methylprednisolone equivalent to 1 mg/kg/day) was administered on day 46. A decrease in the peripheral blood eosinophil count was subsequently observed (Fig. 2), and computed tomography on day 57 showed improvement in the pericardial fluid (Fig. 1C). Based on the course of these events, he was diagnosed with cardiac tamponade associated with eosinophilia. The patient was discharged on day 81.

## 3. Discussion

Here, we report a case of cardiac tamponade due to eosinophilia, probably caused by cGvHD. The patient developed hypotension and required emergency treatment, without awaiting a definitive diagnosis. The patient had an unusual course; however, after careful assessment and treatment, good outcome was achieved.

In this case, eosinophilia was considered the cause of cardiac tamponade. Typical differentiators of cardiac tamponade include pericardial disease, trauma, and cardiac rupture.^[1]^ Pericardial disease is particularly challenging to diagnose because it can be affected by all disease categories, including infectious, neoplastic, autoimmune, inflammatory, metabolic, drug-induced, and congenital disease.^[3]^ A review of patients who underwent echocardiography reported that 3.5% of the patients with systemic disease had pericardial effusion. The causes were as follows: 59.7% had uremia, 15.8% had lymphatic drainage disorders, such as heart failure and cirrhosis, 7.9% had autoimmune disease, 6.8% had neoplastic disease, and 5% had infectious disease.^[4]^ In this case, electrocardiography, blood tests, or imaging studies did not confirm the diagnosis. Pericardial effusions progressed while renal and cardiac function recovered. Pericardial biopsy is useful for definitive diagnosis; however, it was not performed because the blood pressure and coagulation function were not adequately stable. Pericarditis is a rare symptom of cGvHD^[5]^; however, the patient had no other symptoms indicative of recurrent cGvHD on the skin, eyes, or mouth.^[6]^ In the present case, no histopathological diagnosis of myocarditis or pericarditis was made; however, the condition was presumed to be similar to that of eosinophilic myocarditis.

Eosinophilia is observed in patients with various inflammatory and allergic diseases, as well as in patients with various hematologic malignancies. The incidence and prevalence of eosinophilia are not well described.^[7]^ Eosinophilia has been classified as mild (0.5–1.5 × 10^9^/L), marked (>1.5 × 10^9^/L), or massive (>5.0 × 10^9^/L).^[8]^ Although the absolute number of eosinophils did not correlate with the degree or risk of organ damage,^[9]^ this case showed mild-to-marked eosinophilia.

In this case, cGvHD was suspected as the cause of eosinophilia; however, a definitive diagnosis could not be made. Common causes of eosinophilia include parasitic, allergic, autoimmune, connective tissue, and rheumatic diseases.^[8]^ Based on the overproduction of cytokines, such as interleukin-4 and interleukin-5, cGvHD may be a Th-2-mediated process^[10]^; moreover, an association between increased eosinophils and cGvHD has been noted.^[11]^ Screening for autoimmune diseases yielded negative results, and no apparent reactive cause was identified. Fip1-like 1-platelet-derived growth factor receptor alpha and platelet-derived growth factor receptor beta 5q33 translocations, which are genetic abnormalities indicative of myeloproliferation, were negative in this case; moreover, no findings of neoplastic eosinophilia were observed.

Eosinophilia treatment depends on the cause. Severe organ dysfunction, particularly cardiac and pulmonary dysfunction, requires urgent treatment. Early treatment with a corticosteroid can prevent fatal outcomes in cases of eosinophilic myocarditis; however, this has not been adequately verified.^[12]^ In this case, although myocarditis was not diagnosed, emergency treatment with corticosteroid was successful and resulted in favorable outcomes.

## 4. Conclusion

Here, we report a case of cardiac tamponade due to eosinophilia. The diagnosis was challenging owing to the rarity of the mechanism; however, emergency corticosteroid administration was effective, and the patient demonstrated good outcomes. Eosinophilia resulting in cardiac tamponade points to an important differential diagnosis.

## Acknowledgments

We would like to thank Editage (www.editage.com) for English language editing. We are also grateful to our patient for providing consent for writing and publishing this report.

## Author contributions

**Writing – original draft:** Hiroaki Taniguchi.

**Writing – review & editing:** Mayuko Kaneko, Nobuaki Kiriu, Tetsuro Kiyozumi.
